# Nationwide trends in incidence and in-hospital mortality of ARDS in Germany, 2008–2022

**DOI:** 10.1038/s41598-026-69229-4

**Published:** 2026-09-03

**Authors:** Ulrike Heinicke, Thomas Jasny, Jan Andreas Kloka, Martin Scharffenberg, Jakob Wittenstein, Andreas von Knethen, Kai Zacharowski, Benjamin Friedrichson

**Affiliations:** 1 Department of Anesthesiology, Intensive Care Medicine and Pain Therapy, Goethe University Frankfurt, University Hospital Frankfurt, Theodor-Stern-Kai 7, 60590 Frankfurt Am Main, Germany; 2 Pulmonary Engineering Group, Faculty of Medicine and University Hospital Carl Gustav Carus, TU Dresden University of Technology, Dresden, Germany

**Keywords:** Aged, Organ dysfunction, Obesity, COVID-19, Germany, ARDS, Diseases, Health care, Medical research

## Abstract

Acute respiratory distress syndrome (ARDS) remains a major contributor to morbidity and mortality among critically ill patients. Recent events such as the COVID-19 pandemic and demographic changes have likely influenced its epidemiology. This study aimed to investigate nationwide trends in ARDS incidence and in-hospital mortality among adult intensive care patients in Germany over a 15-year period and to describe demographic and clinical differences between survivors and non-survivors. We conducted a retrospective nationwide cohort study using hospital discharge data from the German Federal Statistical Office. All adult patients diagnosed with ARDS (ICD-10 code J80) and treated in intensive care units between 2008 and 2022 were included. Among 87,488 patients, 52.2% died during hospitalization. The incidence increased significantly before the pandemic, peaking at 21.0 per 100,000 inhabitants in 2021. In-hospital mortality declined from 54.2% in 2008 to 50.8% in 2018, followed by an increase up to 53.8% in 2021 and a slight decline thereafter. Non-survivors were older (68 vs 60 yr), had a greater comorbidity burden (Elixhauser index 17 vs 12), and more severe ARDS (54.2% vs 41.1%). Mortality was highest among patients aged 90 to 94 years (82%). COVID-19–related cases had a higher observed in-hospital mortality (53.5% vs 51.8%) and a higher proportion of severe ARDS (77.2% vs 38.6%). ARDS incidence and in-hospital mortality remain high. The low detection of mild to moderate ARDS highlights the need for better diagnostic tools and standardized criteria. Elderly patients are especially vulnerable, underscoring the need for age-specific therapies and improved early detection.

**Supplementary Information:**

The online version contains supplementary material available at https://doi.org/10.1038/s41598-026-69229-4.

## Introduction

The Acute Respiratory Distress Syndrome (ARDS) is a life-threatening condition characterized by acute hypoxemic respiratory failure and diffuse alveolar damage. The clinical diagnosis of ARDS is based on impaired oxygenation, bilateral pulmonary infiltrates, and the exclusion of cardiogenic edema. ARDS frequently occurs in the setting of systemic inflammatory response syndrome (SIRS) and is often associated with multiorgan failure.

The reported incidence of ARDS varies widely between studies. In Europe, estimates range from 3 to 7 cases per 100,000 inhabitants annually^1^, whereas a large prospective cohort study from King County, Washington (USA), reported a crude incidence of 78.9 per 100,000 person-years in 2005^1,2^. This heterogeneity likely reflects differences in ARDS definitions (e.g., American–European Consensus Conference vs. Berlin definition), study populations (e.g., age, comorbidities, disease severity), and local treatment strategies. Moreover, the incidence of ARDS is uniquely influenced by its diagnostic criteria, which are tightly linked to the use of specific interventions, such as positive end-expiratory pressure (PEEP) and high inspiratory oxygen concentrations (FiO₂), thereby affecting study populations and therapeutic decision-making^3–5^. Similarly, estimates of mortality vary widely, typically ranging from 40 to 50%, depending on patient age, comorbidities, ARDS severity, main diagnosis, and the type of study (observational vs. interventional) or time of survey (e.g., 28-day vs. 90-day mortality)^3^.

The Berlin definition from 2012 enabled improved stratification of ARDS severity based on the PaO₂/FiO₂ ratio^6^. One of the first large-scale studies that first adopted this new definition was the international LUNG SAFE study, which found that 10.4% of all ICU patients and 23.4% of ventilated patients met the criteria for ARDS, with severity-dependent mortality ranging from 34 to 46%^7^. Importantly, ARDS remained underdiagnosed by clinicians, suggesting that real-world mortality may be even higher.

Since late 2019, the emergence of severe acute respiratory syndrome coronavirus 2 (SARS-CoV-2) has led to one of the largest pandemics in history. As of August 2025, the World Health Organization (WHO) reported over 778 million confirmed COVID-19 cases and more than 7 million deaths worldwide^8^. Following a period of relative decline, COVID-19 cases and ICU admissions have again increased globally since August 2024^9^.

To date, it remains unclear how the Berlin definition, increasing clinical awareness following the LUNG SAFE study, and the COVID-19 pandemic have affected national trends in ARDS incidence and mortality. Therefore, the aim of this study was to evaluate long-term trends in ARDS incidence and in-hospital mortality in Germany from 2008 to 2022, and to identify clinical characteristics associated with in-hospital survival.

## Results

### Demographic and clinical characteristics

The study population included all adult (≥ 18 years) ARDS patients admitted to an ICU in Germany between 2008 and 2022, as recorded by the German Federal Statistical Office (87,488 cases). Of these ARDS patients, 41,816 (47.8%) survived ARDS (survivors) and 45,672 (52.2%) did not survive ARDS (non-survivors; Table 1). Men accounted for a larger proportion of ARDS cases than women but there were no significant differences in sex distribution between survivors and non-survivors (34.1% vs 33.6% females; p = 0.155, respectively). Median age (60, [49–70] vs 68 [58–77] yr; p < 0.0001) and the Elixhauser (EH) comorbidity index (12^5–19^ vs 17^10–24^; p < 0.0001) were lower in survivors than in non-survivors. Survivors had longer observed hospital length of stay (LOS) (31.4 [18–51] vs 14.7 [7–27] d; p < 0.0001) and ICU stay (19 [9–33] vs 10^4–20^ d; p < 0.0001) than non-survivors. These comparisons should be interpreted in light of earlier death among non-survivors, which limits the observed duration of hospitalization and intensive care treatment.

**Table 1:** Characteristics of the ARDS Cohort.

	survivors n [%]	non-survivors n [%]	P Value
Total	41,816 (47.8)	45,672 (52.2)	
Sex, F	14,239 (34.1)	15,344 (33.6)	0.155
Age, yr	60 (49–70)	68 (58–77)	<.0001
EH index	12 (5–19)	17 (10–24)	<.0001
Hospital LOS, d	31.4 (18–51)	14.7 (7–27)	<.0001
ICU stay, d	19 (9–33)	10 (4–20)	<.0001
ARDS severity, 2015–2022 only^#^			
mild	2,359 (5.64)	1,126 (2.47)	<.0001
moderate	8,813 (21.1)	5,663 (12.4)	<.0001
severe	17,166 (41.1)	24,745 (54.2)	<.0001
unspecific	1,576 (3.77)	1,390 (3.04)	<.0001
ARDS main diagnosis*			
Extrapulmonary sepsis	22,417 (53.6)	30,757 (67.3)	<.0001
Pneumonia	25,473 (60.9)	27,031 (59.2)	<.0001
COVID-19	9,,785 (23.4)	11,,263 (24.7)	<.0001
Aspiration pneumonia	4,,417 (10.6)	4,608 (10.1)	0.021
Trauma	1,322 (3.16)	559 (1.22)	<.0001
Pancreatitis	1,012 (2.42)	1,085 (2.38)	0.667
Drown	51 (0.12)	63 (0.14)	0.513
Comorbidity			
Congestive heart failure	11,101 (26.6)	15,655 (34.3)	<.0001
Hypertension	21,751 (52.0)	21,831 (47.8)	<.0001
Chronic pulmonary disease	6,850 (16.4)	7,284 (16.0)	0.082
Diabetes	12,281 (29.4)	13,690 (30.0)	0.050
Renal failure	6,665 (15.9)	9,571 (21.0)	<.0001
Malignancy	2,824 (6.8)	5,420 (11.9)	<.0001
Obesity	7,184 (17.2)	5,746 (12.6)	<.0001
Ventilation			
Ventilation, yes	39,999 (95.7)	44,638 (97.7)	<.0001
Ventilation, d	15.5 (6.9–28.8)	9.6 (3.3–19.0)	<.0001
Assist device			
ECMO-VV	4,280 (10.2)	5,995 (13.1)	<.0001
ECMO-VA	572 (1.37)	1,358 (2.97)	<.0001
PECLA	421 (1.01)	603 (1.32)	<.0001
ECOO2R	61 (0.15)	127 (0.28)	<.0001
Microaxial pump	50 (0.12)	107 (0.23)	<.0001

Data are presented as n (%) for categorical variables or median (interquartile range) for continuous variables. Group differences between categorical variables were tested for statistical significance with the χ2 test, and for continuous variables with Wilcoxon rank-sum test.^#^Percentages for ARDS severity are calculated within the subgroup with available Berlin severity coding (2015–2022); cases from 2008–2014 are excluded from this denominator because no Berlin severity coding was available. *The different codes for the main diagnosis of ARDS were not mutually exclusive and were collected during the ICU stay involving ARDS. ECMO-VA, venoarterial extracorporeal membrane oxygenation; ECMO-VV, venovenous extracorporeal membrane oxygenation; ECOO2R, Extracorporeal Carbon Dioxide Removal; PECLA, Pumpless Extracorporeal Lung Assist.

Pre-existing comorbidities such as congestive heart failure (26.6% vs 34.3%; p < 0.0001), diabetes (29.4% vs 30%; p = 0.050), renal failure (15.9% vs 21.0%; p < 0.0001), and malignancy (6.8% vs 11.9%; p < 0.0001) were more frequent among non-survivors than among survivors (Table 1). In contrast, hypertension (52.0% vs 47.8%; p < 0.0001) and obesity (17.2% vs 12.6%; p < 0.0001) were more frequent among survivors.. Further components of the Elixhauser comorbidity index are presented in Supplement Table 1.

Invasive mechanical ventilation was more frequently used in non-survivors than in survivors (95.7% vs. 97.7%; p < 0.0001). Likewise, the use of extracorporeal support devices (venovenous extracorporeal membrane oxygenation (ECMO-VV), venoarterial ECMO (ECMO-VA), Pumpless Extracorporeal Lung Assist (PECLA), Extracorporeal Carbon Dioxide Removal (ECCO2R), and microaxial pumps) were more frequently used in non-survivors (all p < 0.0001). The observed duration of invasive ventilation was longer in survivors than in non-survivors (15.5 [6.9–28.8] vs 9.6 [3.3–19.0] d; p < 0.0001). These comparisons should be interpreted cautiously because earlier death among non-survivors limits the observed duration of ventilation.

### Complications

The most common complications in both the survivor and the non-survivor group were renal replacement therapy (26.8% vs 49.2%; p < 0.0001) and cardiopulmonary resuscitation (CPR, 9.7% vs. 22.8%; p < 0.0001), which occurred twice as often in non-survivors compared to survivors of ARDS (Table 2). This was followed by pulmonary embolism (6.5% vs 6.7%; p = 0.1796), which displayed no statistically significant difference between both groups. Furthermore, myocardial infarction (3.4% vs 4.8%; p < 0.0001), ischaemic stroke (3.4% vs 4.1%; p < 0.0001), and intracerebral bleeding (ICB, 2.3% vs 3.9%; p < 0.0001) were significantly more frequent in the non-survivors compared with the survivors group. Further complications are depicted in Table 2.

**Table 2:** Complications and discharge of the ARDS Cohort.

	**survivors** **n [%]**	**non-survivors** **n [%]**	***P* Value**
complications			
Renal replacement therapy	11,197 (26.8)	22,469 (49.2)	<.0001
CPR	4,036 (9.7)	10,394 (22.8)	<.0001
Pulmonary embolism	2,711 (6.5)	3,064 (6.7)	0.180
Myocardial infarction	1,440 (3.4)	2,174 (4.8)	<.0001
Ischaemic Stroke	1,406 (3.4)	1,865 (4.1)	<.0001
Intracerebral bleeding	954 (2.3)	1,794 (3.9)	<.0001
Arterial embolism/thrombosis	768 (1.8)	1,667 (3.7)	<.0001
Anoxic brain damage	676 (1.6)	1,364 (3.0)	<.0001
Cerebral oedema	609 (1.5)	1,168 (2.6)	<.0001
CPR prior to hospitalisation	425 (1.0)	824 (1.8)	<.0001
discharge			
Home	17,166 (41.1)	-	-
Transfer	16,712 (40.0)	-	-
Rehab	6,898 (16.5)	-	-
Care	993 (2.4)	-	-
Hospice	45 (0.1)	-	-
Death	-	45,672 (100)	-

Data are presented as n (%) for categorical variables. Percentages for discharge categories are calculated based on survivors only, whereas complication rates are calculated based on the respective group size (survivors or non-survivors). Group differences between categorical variables were tested for statistical significance with the χ2 test. CPR, Cardiopulmonary resuscitation.

In addition, most of the survivors were discharged to home (41.1%), followed by transfer (40.0%), rehabilitation (16.5%), care (2.4%), and hospice (0.1%; Table 2).

### ARDS severity classification and main diagnosis

**Fig. 1:**
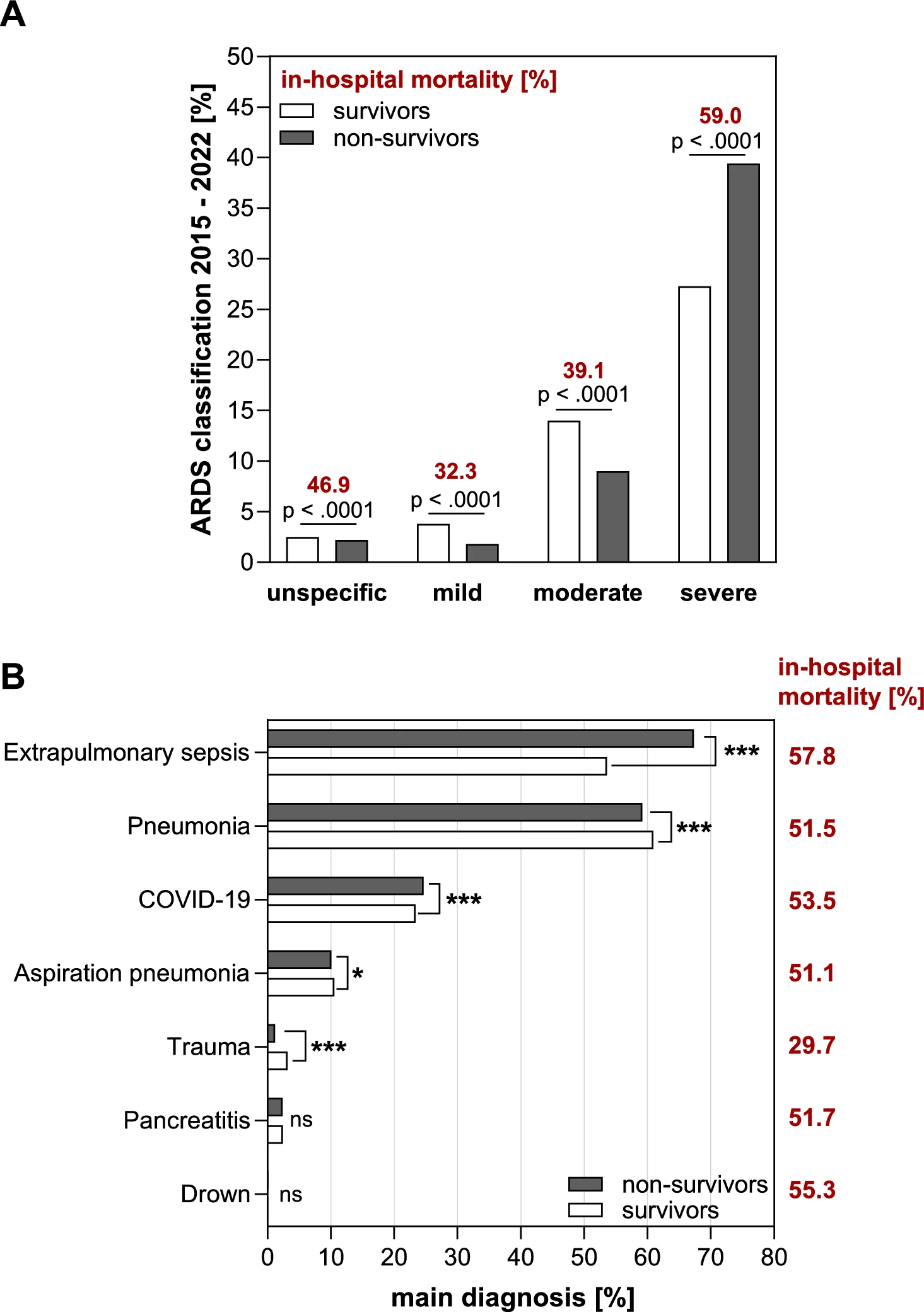
ARDS classification and main diagnosis. A, The ARDS classification according to the 2012 Berlin definition was introduced into the German ICD coding system in 2015. Therefore, the categories unspecific, mild, moderate, and severe ARDS are shown only for 2015–2022. Percentages indicate the proportion of severity-coded ARDS cases within the 2015–2022 subgroup, and the red values show in-hospital mortality within each severity category. B, Main diagnosis of survivors and non-survivors of ARDS with in-hospital mortality in percent is depicted. The different codes for the main diagnosis of ARDS were not mutually exclusive and were collected during the ICU stay involving ARDS.

The ARDS classification based on the Berlin ARDS definition from 2012, which was introduced into the ICD coding system in Germany in 2015. Unspecific ARDS cases occurred and were significantly more frequent in survivors compared to non-survivors (3.77% vs 3.04%; p < 0.0001). Additionally, mild and moderate ARDS cases occurred more often in survivors compared to non-survivors (mild: 5.64% vs 2.47%; p < 0.0001; moderate 21.1% vs 12.4%; p < 0.0001). In contrast, severe ARDS cases were more frequently in non-survivors compared to survivors of ARDS (54.2% vs. 41.1%; p < 0.0001) and accounted for the largest proportion of all ARDS cases in both groups (Fig. 1A; Table 1). In-hospital mortality increased with severity of ARDS and was highest with 59.0% in severe ARDS (Fig. 1A; Supplement Table 3).

**Table 3:** ARDS case distribution and in-hospital mortality over time.

time	total	survivors	non-survivors	mortality rate
[yr]	n [%]	n [%]	n [%]	[%]
2008	3,394 (3.88)	1,555 (3.73)	1,839 (4.03)	54.2
2009	3,595 (4.11)	1,720 (4.11)	1,875 (4.11)	52.2
2010	3,556 (4.06)	1,703 (4.07)	1,853 (4.06)	52.1
2011	3,842 (4.39)	1,920 (4.59)	1,922 (4.21)	50.0
2012	3,541 (4.05)	1,744 (4.17)	1,797 (3.93)	50.7
2013	3,936 (4.50)	1,959 (4.68)	1,977 (4.33)	50.2
2014	3,835 (4.38)	1,852 (4.43)	1,983 (4.34)	51.7
2015	4,649 (5.31)	2,248 (5.38)	2,401 (5.26)	51.6
2016	5,180 (5.92)	2,567 (6.14)	2,613 (5.72)	50.4
2017	5,111 (5.84)	2,509 (6.00)	2,602 (5.70)	50.9
2018	5,715 (6.53)	2,813 (6.73)	2,902 (6.35)	50.8
2019	5,603 (6.40)	2,685 (6.42)	2,918 (6.39)	52.1
2020	9,467 (10.82	4,440 (10.62)	5,027 (11.01)	53.1
2021	17,499 (20.00)	8,089 (19.34)	9,410 (20.60)	53.8
2022	8,565 (9.79)	4,012 (9.59)	4,553 (9.97)	53.2

Data are presented as n (%) for categorical variables.

Two of the most frequent main diagnoses in survivors and non-survivors of ARDS were extrapulmonary sepsis (53.6% vs 67.3%; p < 0.0001) and COVID-19 (23.4% vs 24.7%; < 0.0001), which occurred significantly more often in non-survivors compared to survivors of ARDS (Fig. 1B; Table 1). In contrast, pneumonia (60.9% vs 59.2%; < 0.0001), aspiration pneumonia (10.6% vs 10.1%; p = 0.021), and trauma (3.16% vs 1.22%; p < 0.0001) occurred more frequently in survivors compared to non-survivors of ARDS. Pancreatitis (2.42% vs 2.38%; p = 0.667) and drowning (0.12% vs 0.14%; p = 0.513) showed no significant differences between survivors and non-survivors of ARDS (Fig. 1B; Table 1). Thereby, the in-hospital mortality was highest with extrapulmonary sepsis (57.8%), followed by drowning (55.3%), COVID-19 (53.5%), pancreatitis (51.7%), pneumonia (51.5%), aspiration pneumonia (51.1%), and trauma (29.7%; Fig. 1B, Supplement Table 4).

**Table 4:** ARDS age distribution and in-hospital mortality.

age	total	survivors	non-survivors	mortality rate
yr	n [%]	n [%]	n [%]	[%]
18–19	451 (0.52)	330 (0.79)	121 (0.26)	26.8
20–24	1,259 (1.44)	919 (2.20)	340 (0.74)	27.0
25–29	1,490 (1.70)	1,096 (2.62)	394 (0.86)	26.4
30–34	2,038 (2.33)	1,415 (3.38)	623 (1.36)	30.6
35–39	2,617 (2.99)	1,768 (4.23)	849 (1.86)	32.4
40–44	3,609 (4.13)	2,367 (5.66)	1,242 (2.72)	34.4
45–49	5,285 (6.04)	3,280 (7.84)	2,005 (4.39)	37.9
50–54	7,538 (8.62)	4,483 (10.72)	3,055 (6.69)	40.5
55–59	9,420 (10.77)	5,096 (12.19)	4,324 (9.47)	45.9
60–64	10,722 (12.26)	5,329 (12.74)	5,393 (11.81)	50.3
65–69	11,305 (12.92)	5,040 (12.05)	6,265 (13.72)	55.4
70–74	11,000 (12.57)	4,382 (10.48)	6,618 (14.49)	60.2
75–79	10,313 (11.79)	3,594 (8.59)	6,719 (14.71)	65.2
80–84	7,307 (8.35)	2,042 (4.88)	5,265 (11.53)	72.1
85–89	2,618 (2.99)	579 (1.38)	2,039 (4.46)	77.9
90–94	455 (0.52)	82 (0.20)	373 (0.82)	82.0
95–110	61 (0.07)	14 (0.03)	47 (0.10)	77.0

Data are presented as n (%) for categorical variables.

### Incidence and in-hospital mortality of ARDS in Germany

**Fig. 2:**
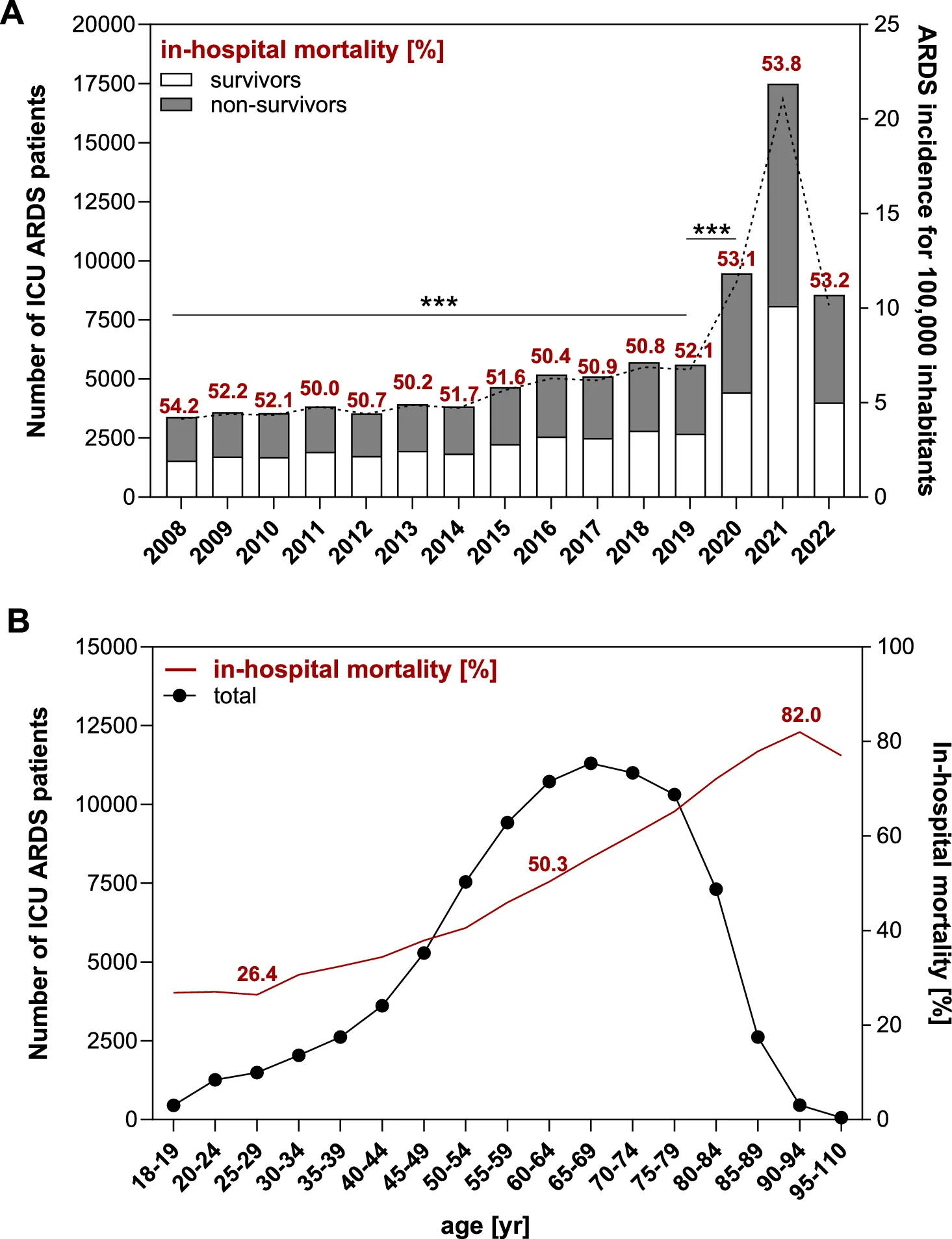
ARDS incidence, age distribution and in-hospital mortality A, Number of survivors and non-survivors of ICU ARDS patients and ARDS incidence for 100,000 inhabitants over time with in-hospital mortality in percent is depicted. B, Number of total ICU ARDS patients and in-hospital mortality of ARDS with age is depicted. ***p ˂ 0.001.

The number of ICU ARDS patients in Germany increased slightly over time, with noticeable increases between 2014 to 2015 (4.38% to 5.31%) and from 2019–2021 (2019: 6.4%, 2020: 10.8%, 2021: 20.0%). In 2022, the number of ICU ARDS patients declined to 8.565 (9.8%; Table 3; Fig. 2A). Accordingly, the incidence of ARDS for 100,000 inhabitants increased slightly over time from 4.1 (2008) to 6.9 (2018) and has its peak with 21.0 in 2021. Thereby, it stagnated at a relatively stable plateau between the years 2008–2014, then rose almost continuously from 2015–2018, before it extremely increased during the COVID-19 pandemic, and fall down abruptly in 2022 to a relatively high level (Fig. 2A; Table 3; Supplement Table 2). Interestingly, despite the increasing incidence of ARDS for 100,000 inhabitants, the in-hospital mortality of ARDS decreased over time from 54.2% (2008) to 50.8% (2018) followed by an increase to 53.8% in 2021 (Table 3, Fig. 2A). Notably, in 2019 the number of ICU ARDS patients (5,603) and the incidence of ARDS for 100,000 inhabitants (6.7) slightly decreased, whereas in-hospital mortality increased (52.1%; Table 3, Fig. 2A; Supplement Table 2). To evaluate significant changes in ARDS incidence and in-hospital mortality over time, we employed an interrupted time series (ITS) model with a generalized least squares (GLS) approach. The model demonstrated a statistically significant increasing trend in ARDS incidence prior to the COVID-19 pandemic (estimate = + 0.26 per year, p < 0.001), as well as a significant level increase at the onset of the pandemic (estimate = + 7.04, p < 0.001). However, no significant change in slope was observed in the post-COVID period (estimate = + 0.64 per year, p = 0.437; see Supplement Table 9). These results show a marked increase in ARDS incidence in 2020. However, because only three post-pandemic annual observations were available, conclusions regarding subsequent trend stabilization remain uncertain. For in-hospital mortality, the model revealed no statistically significant trend before the COVID-19 pandemic (estimate = –0.14 per year, p = 0.25), no significant level change in 2020 (estimate = + 1.89, p = 0.28), and no significant trend change afterward (estimate = + 0.37 per year, p = 0.64; see Supplement Table 10). No statistically significant change in annual in-hospital mortality trends was detected around the onset of the pandemic. However, the limited number of post-pandemic observations substantially reduced the power to detect trend changes, and these findings should therefore be interpreted cautiously.

### ARDS age distribution

The age distribution of adult ARDS ICU patients demonstrated that ARDS mainly occurred in elderly and had its peak with 11,305 (12.93%) cases at 65–69 yr (Table 4, Fig. 2B). However, survivors of ARDS predominated among younger patients (18–64 yr), whereas among older patients (65–110 yr) the proportion of non-survivors of ARDS predominated (Table 4, Fig. 2B), resulting in an age peak of ARDS ICU patients at 60–64 yr (12.74%) for survivors, and at 75–79 yr (14.71%) for non-survivors of ARDS. Notably, the in-hospital mortality of ARDS increased with age, with the lowest in-hospital mortality (26.4%) with an age of 25–29 yr, reaching 50.3% with an age of 60–64 yr and had its peak (82.0%) with an age of 90–94 yr (Table 4, Fig. 2B).

### Comparison of characteristics between COVID-19 and non-COVID-19 ARDS

Of the 87,488 patients included in the analysis, 21,048 (24.1%) were diagnosed with COVID-19-associated ARDS (CARDS; Supplementary Table 5). The proportion of female patients was lower in the COVID-19 cohort compared with the non-COVID ARDS cohort (32.0% vs. 34.4%; p < 0.0001), and the median age was significantly higher (66 [56–75] vs. 64 [52–74] years; p < 0.0001). The age distribution indicated that CARDS predominantly affected older individuals and was more frequently observed among patients aged 55–89 years (Supplementary Table 6). Although CARDS was associated with higher in-hospital mortality compared with non-COVID ARDS in our cohort, this patient-level difference does not necessarily translate into a detectable change in overall ARDS mortality trends at the population level. The ITS analysis evaluates temporal changes across all ARDS cases, including changes in case mix, treatment strategies, and the relative proportion of CARDS cases over time. Notably, the EH index (11^5–18^ vs. 15^8–23^; p < 0.0001), hospital length of stay (19.9 [11–34] vs. 22.7 [11–41] d; p < 0.0001), and ICU stay (14 [7–25] vs. 14 [5–26] d; p < 0.0001) were significantly shorter in the COVID-19 cohort compared to the non-COVID-19 cohort (Supplementary Table 5).

While mild and unspecific ARDS cases were more prevalent in the non-COVID-19 cohort, moderate and severe ARDS cases were significantly more common in the COVID-19 cohort (Supplementary Table 5). In particular, severe ARDS cases were twice as frequent in the COVID-19 cohort (77.2%) compared to the non-COVID-19 cohort (38.6%). Moreover, all primary main diagnoses of ARDS (excluding COVID-19) were significantly less frequent in the COVID-19 cohort than in the non-COVID-19 cohort.

As previously reported, pre-existing comorbidities such as hypertension (59.7% vs. 46.7%; p < 0.0001), diabetes (34.9% vs. 28.0%; p < 0.0001), and obesity (19.4% vs. 13.3%; p < 0.0001) were significantly more common in the COVID-19 cohort compared to the non-COVID-19 cohort.

Ventilation was required significantly more often in the non-COVID-19 cohort than in the COVID-19 cohort (96.9% vs. 96.4%; p = 0.0003). However, the duration of ventilation was significantly longer in the COVID-19 cohort (12.4^6–24^ d) compared to the non-COVID-19 cohort (11.9^5–24^ d; p < 0.0001). Notably, the use of assist devices such as ECMO-VV (15.9% vs. 10.4%; p < 0.0001) and ECCO2R (0.4% vs. 0.2%; p < 0.0001) was significantly higher in the COVID-19 cohort, whereas other assist devices (ECMO-VA, PECLA, and microaxial pumps) were used more frequently in the non-COVID-19 cohort (Supplementary Table 5).

Interestingly, pulmonary embolism (12.3% vs. 4.8%; p < 0.0001) and intracerebral bleeding (3.6% vs. 3.1%; p = 0.0007) were significantly more common in the COVID-19 cohort compared to the non-COVID-19 cohort. Additional complications are presented in Supplementary Table 7.

Furthermore, a higher proportion of CARDS patients were discharged home compared to non-COVID-19 patients (20.7% vs. 19.3%). In-hospital mortality was also significantly higher in the COVID-19 cohort (53.5%) compared to the non-COVID-19 cohort (51.8%; p < 0.0001).

## Discussion

This retrospective nationwide study provides new insights into the incidence and in-hospital mortality of ARDS among adult ICU patients in Germany over a 15-year period, including the critical period of the COVID-19 pandemic. Our key findings highlight several important trends.

First, we observed a steady increase in the incidence of ARDS per 100,000 inhabitants, peaking in 2021 during the COVID-19 pandemic. In contrast, in-hospital mortality decreased over time from 2008 to 2018, before rising again during the COVID-19 period. Importantly, we found that survival of ARDS were younger, had a lower EH index, and more often had hypertension and obesity. The study also revealed a high in-hospital mortality of ARDS in patients suffering from extrapulmonary sepsis, and in ARDS patients of older age.

As previously described, CARDS patients were predominantly older, male, and more likely to have pre-existing conditions such as hypertension, diabetes, and obesity^10,11^. Moreover, CARDS had a higher observed in-hospital mortality rate (53.5%) compared to non-COVID-19 ARDS.

These results are largely in line with previously published data from other countries (Supplement Table 8). A recent large-scale study in Spain analysed mechanically ventilated ARDS (MV-ARDS) from 2000 to 2022. Similar to our results, the incidence of ARDS peaked in 2021 during the COVID-19 pandemic, and hospital mortality decreased over time, although it remained high during the pandemic period^12^. Although, our study focused on a broader cohort of ARDS patients in Germany, including those with both invasive and non-invasive treatments. Furthermore, the international LUNG SAFE study from 2016 found a severity-dependent mortality of 34–46%, which is similar to the severity-dependent mortality in our study (32–59%)^7^. However, in-hospital mortality of severe ARDS ICU patients in our study was higher (59%), which can probably be attributed to the unusually high proportion of severe ARDS patients during COVID-19. Furthermore, a nationwide analysis from 2017 of 12,846 adult ARDS ICU patients in France revealed a mortality of 51.1%, which is similar to the in-hospital mortality we found in 2017 (50.9%)^13^. In addition, a currently published study from Karagiannidis et al. evaluated 1,003,882 patients, who were ventilated in Germany between 2019 and 2022. The authors observed also a steady increase in mortality with age, especially in patients of 60 years and older^14^. Since we analysed all ARDS ICU patients, the in-hospital mortality in our study is noticeably higher.

Interestingly, in our study, hypertension and obesity were more frequent among survivors of ARDS. However, these findings should be interpreted as descriptive and hypothesis-generating, because the study did not perform multivariable adjustment and residual confounding by age, case mix, comorbidity burden, and disease severity cannot be excluded. The relationship between obesity and mortality in ARDS patients remains incompletely understood and is often discussed in the context of the “obesity paradox^15–18^.

Interestingly, we reported a relatively high number of ARDS patients that received extracorporeal life support (ECLS; 12.9% in the survivors, 17.9% in the non-survivors group) which is substantially higher than in the French study of Papazian et al. from 2021, which reported that only 6.1% of the ARDS patients received ECLS^13^. However, this finding is consistent with previous reports, including our earlier COVID-19–related analysis^19–21^. In addition, we reported in this study a relatively high rate of CPR with 9.7% in the survivors and 22.8% in the non-survivors group of ARDS. We previously reported in a cohort of hospitalized COVID-19 patients, a CPR incidence of 1.73% among all inpatients and 14.23% among non-surviving ICU patients^17^. However, this study included only adult ICU patients, many of whom were of advanced age and had prolongedhospital LOS, reflecting a cohort with substantial disease burden that may not be fully comparable to younger or less severely affected ARDS populations.

This study had several limitations. First, ARDS is a heterogeneous syndrome diagnosed on the basis of clinical criteria, and reliance on ICD coding may have introduced variability in case ascertainment. Although ICD coding linked to the German reimbursement system provides a high level of coding accuracy, mild and moderate ARDS may have been underrepresented. The relatively high mortality observed even among patients coded as mild ARDS should therefore be interpreted in the context of this selected cohort, which consisted exclusively of ICU patients with a high proportion of mechanically ventilated patients and substantial comorbidity burden. In addition, some patients receiving non-invasive ventilation or high-flow oxygen therapy may have been treated outside the ICU, potentially leading to further underrepresentation of less severe cases. The new Global ARDS definition (2023) may improve case detection in the future^22^.

Second, administrative databases inherently carry risks of misclassification, selection bias, and limited clinical detail, as important physiological variables and treatment-related information are not available^23^. Consequently, the comparisons between survivors and non-survivors were descriptive and unadjusted, and no conclusions regarding independent associations with in-hospital mortality can be drawn. Given the large sample size and the number of comparisons performed, small absolute differences may achieve statistical significance and should be interpreted cautiously.

Third, temporal changes in ARDS definitions and coding practice may have influenced case ascertainment over time. The Berlin Definition was introduced in 2012 and implemented in German ICD coding in 2015, which may have affected the identification and classification of ARDS cases during the study period.

Finally, the interrupted time-series analysis was based on annual data with only three post-pandemic observations, limiting power to detect post-COVID trend changes. Moreover, the MA(1) parameter of the incidence model was located at the invertibility boundary, indicating potential model instability and warranting cautious interpretation of the ITS results. Despite these limitations, this nationwide dataset provides valuable epidemiological insights into ARDS in German ICUs. The key findings emphasise the increase in ARDS incidence during the pandemic and the subsequent rise in mortality due to the higher proportion of severe cases. The identification of age, comorbidities, and disease severity as important factors in ARDS outcomes reinforces the need for targeted interventions. Moving forward, further studies should focus on improving early detection and optimal management strategies, particularly for mild and moderate ARDS cases, which are currently underrepresented in clinical data. Additionally, the long-term effects of the COVID-19 pandemic on ARDS incidence and outcomes should be closely monitored to inform future public health strategies.

## Methods

### Study design and data source

We conducted a retrospective, nationwide cohort study including all adult patients (≥ 18 years) with ARDS patients admitted to ICUs in public, private, and university/teaching hospitals across Germany from 2008 to 2022. In Germany, all hospitals are legally required to report diagnoses using the International Statistical Classification of Diseases and Related Health Problems (ICD) 10th edition and International Statistical Classification of Procedures (OPS) codes to the Institute for invoice system within hospitals (InEK)^24^. Reimbursement data are transferred to the German Federal Statistical Office and made available to researchers under strict data use agreements. Patient data are stored locally and accessible only via remote aggregated queries, ensuring complete anonymity of patients and hospitals. To date, no validation study has been conducted using these specific data sources.

### Study population and variables

ARDS was identified using ICD-10 code J80. From 2015 onward, ARDS severity was additionally classified as mild, moderate, or severe according to the Berlin Definition using the ICD-10-GM subcodes J80.01 (mild), J80.02 (moderate), J80.03 (severe), and J80.09 (unspecific)^6^, based on ICD coding updates. Only the most severe category during the hospital stay was retained for analysis. Collected variables included patient demographics, comorbidities, complications, and in-hospital mortality. The Elixhauser comorbidity index was calculated using established ICD groupings (Supplement Table S1). Comorbidity burden was quantified using the weighted van Walraven Elixhauser comorbidity score, which ranges from − 19 to + 89, with higher values indicating greater comorbidity burden. Each patient was recorded as one hospital case with a non-traceable ID; duplicates from inter-hospital transfers were minimized via cross-checking of key variables (age, sex, admission/discharge dates and type, region). Mortality data refer to in-hospital deaths, as post-discharge follow-up is unavailable.

Administrative data have limitations, such as potential misclassification, lack of clinical detail, and selection bias^23^. To mitigate these, we used validated ICD-10 and OPS codes and relied on legally mandated reporting for reimbursement, ensuring comprehensive nationwide coverage (Supplement Table 11). The Federal Statistical Office applies internal data validation before release. Despite detailed matching and manual checks, some clinical variables (e.g., physiological parameters, treatment specifics) are unavailable, limiting analysis depth. This study adheres to the STROBE guidelines and the Declaration of Helsinki.

### Objectives

The primary aim was to evaluate long-term trends in ARDS incidence and in-hospital mortality in Germany. The secondary aim was to compare demographic and clinical characteristics between survivors and non-survivors..

### Statistical analysis

Categorical variables are presented as counts and percentages; continuous variables are reported as medians with interquartile ranges (IQR). Between-group comparisons were conducted using the χ^2^ test or Wilcoxon rank-sum test, as appropriate. Statistical significance was defined as p < 0.05 (two-sided). ITS regression with GLS and ARMA(1,1) error structure was used to evaluate trends in ARDS incidence and mortality, accounting for autocorrelation and the onset of the COVID-19 pandemic (2020) as an intervention. The model included a continuous time term (Time), a binary indicator for the onset of the COVID-19 pandemic in 2020 (Intervention), and a post-intervention slope term (Post.intervention.time). Model parameters were estimated by maximum likelihood, and the ARMA order was selected according to the Akaike Information Criterion (AIC). Given the limited number of annual observations, particularly after 2020, the ITS analyses were considered exploratory and interpreted cautiously. Statistical analyses were performed using SAS version 9.4M6 (SAS Institute Inc., Cary, NC, USA) and R version 4.4.3 (nlme package). Graphs were generated with GraphPad Prism version 10.0.0 (GraphPad Software, Boston, MA, USA).

## Supplementary Information

Supplementary Information 1.

Supplementary Information 2.

## Data Availability

The data used in this study are available from the Federal Statistical Office (Destatis) upon application and subject to the applicable data access and licensing conditions. The dataset was used under licence for the present study and is therefore not publicly accessible. Aggregated data generated from the licensed dataset are available from the corresponding author on reasonable request.

## Abbreviations

ARDS: Acute respiratory distress syndrome
COVID-19: Coronavirus disease 2019
CPR: Cardiopulmonary resuscitation
ECLS: Extracorporeal Life Support
ECMO-VV: Venovenous extracorporeal membrane oxygenation
ECMO-VA: Venoarterial extracorporeal membrane oxygenation
PECLA: Pumpless Extracorporeal Lung Assist
ECCO2R: Extracorporeal Carbon Dioxide Removal
ICB: Intracerebral bleeding
ICD: International Statistical Classification of Diseases and Related Health Problems
ICU: Intensive care unit
InEK: Institute for invoice system within hospitals
IQR: Inter Quartile Range
LOS: Length of stay
MACCE: Major adverse cardiovascular and cerebrovascular events
OPS: International Statistical Classification of Procedures
PEEP: Positive End-Expiratory Pressure
SARS-CoV-2: Severe acute respiratory syndrome coronavirus type 2
SD: Standard deviation
SIRS: Systemic Inflammatory Response Syndrome
STROBE: Strengthening the Reporting of Observational Studies in Epidemiology
VTE: Venous thromboembolism
WHO: World Health Organization
