# Supplementary material for: Nationwide trends in incidence and in-hospital mortality of ARDS in Germany, 2008–2022
Source: Sci Rep. 2026 Sep 3;16:27667. doi: 10.1038/s41598-026-69229-4 (PMC13542158; doi:10.1038/s41598-026-69229-4)
Supplement: Supplementary file 1 [file 41598_2026_69229_MOESM1_ESM.docx]

**Supplementary Material**

**Supplement Table 1 Components of the Elixhauser comorbidity index**

|  | **survivors** | **non-survivors** | ***P* value** |
| --- | --- | --- | --- |
| **components** | **n [%]** | **n [%]** |  |
| Cardiac arrhythmias | 15.533 (37.2) | 20.972 (45.9) | <.0001 |
| Valvular disease | 3.715 (8.88) | 4.751 (10.4) | <.0001 |
| Pulmonary circulation disorders | 4.729 (11.3) | 5.912 (12.9) | <.0001 |
| Peripheral vascular disorders | 3.945 (9.4) | 5.940 (13.0) | <.0001 |
| Hypertension, uncomplicated | 19.183 (45.9) | 18.696 (40.9) | <.0001 |
| Hypertension, complicated | 3.108 (7.43) | 3.592 (7.9) | 0.0163 |
| Paralysis | 4.154 (9.93) | 2.483 (5.4) | <.0001 |
| Other neurological disorders | 5.317 (12.7) | 5.547 (12.2) | 0.0107 |
| Chronic pulmonary disease | 6.850 (16.4) | 7.284 (16.0) | 0.0823 |
| Diabetes, uncomplicated | 9.702 (23.2) | 10.475 (22.9) | 0.3502 |
| Diabetes, complicated | 2.766 (6.61) | 3.360 (7.36) | <.0001 |
| Hypothyroidism | 5.839 (14.0) | 4.923 (10.8) | <.0001 |
| Renal failure | 6.665 (15.9) | 9.571 (21.0) | <.0001 |
| Liver disease | 5.099 (12.2) | 13.054 (28.6) | <.0001 |
| Peptic ulcer disease excluding bleeding | 181 (0.43) | 168 (0.37) | 0.1276 |
| AIDS/HIV | 203 (0.49) | 202 (0.44) | 0.3473 |
| Lymphoma | 373 (0.89) | 889 (1.95) | <.0001 |
| Metastatic cancer | 1.189 (2.84) | 2.576 (5.64) | <.0001 |
| Solid tumor without metastasis | 1.798 (4.30) | 3.253 (7.12) | <.0001 |
| Rheumatoid arthritis/collagen vascular diseases | 1.047 (2.50) | 1.388 (3.04) | <.0001 |
| Coagulopathy | 15.166 (36.3) | 22.836 (50.0) | <.0001 |
| Obesity | 7.184 (17.2) | 5.746 (12.6) | <.0001 |
| Weight loss | 4.145 (9.91) | 4.083 (8.94) | <.0001 |
| Fluid and electrolyte disorders | 29.409 (70.3) | 33.332 (73.0) | <.0001 |
| Blood loss anemia | 532 (1.27) | 468 (1.02) | 0.0006 |
| Deficiency anemia | 2.149 (5.14) | 1.502 (3.29) | <.0001 |
| Alcohol abuse | 3.489 (8.34) | 3.434 (7.52) | <.0001 |
| Drug abuse | 1.525 (3.65) | 579 (1.27) | <.0001 |
| Psychosis | 837 (2.00) | 328 (0.72) | <.0001 |
| Depression | 3.942 (9.43) | 1.770 (3.88) | <.0001 |

Data are presented as n (%) for categorical variables. Group differences between categorical variables were tested for statistical significance with the χ2 test.

**Supplement Table 2 ARDS incidence and in-hospital mortality over time**

| **time**  **[yr]** | **ICU**  **stays**  **n** | **ARDS**  **cases**  **n** | **incidence for 100.000**  **ICU cases** | **population**  **Germany**  **n** | **incidence for 100.000**  **inhabitants** |
| --- | --- | --- | --- | --- | --- |
| 2008 | 567.532 | 3.394 | 598,03 | 82.002.356 | 4.14 |
| 2009 | 555.951 | 3.595 | 646,64 | 81.802.257 | 4.39 |
| 2010 | 560.204 | 3.556 | 634,77 | 81.751.602 | 4.35 |
| 2011 | 559.326 | 3.842 | 686,90 | 80.327.900 | 4.78 |
| 2012 | 559.154 | 3.541 | 633,28 | 80.523.746 | 4.40 |
| 2013 | 560.465 | 3.936 | 702,27 | 80.767.463 | 4.87 |
| 2014 | 573.518 | 3.835 | 668,68 | 81.197.537 | 4.72 |
| 2015 | 572.438 | 4.649 | 812,14 | 82.175.684 | 5.66 |
| 2016 | 577.076 | 5.180 | 897,63 | 82.521.653 | 6.28 |
| 2017 | 568.793 | 5.111 | 898,57 | 82.792.351 | 6.17 |
| 2018 | 557.874 | 5.715 | 1.024,42 | 83.019.213 | 6.88 |
| 2019 | 556.905 | 5.603 | 1.006,10 | 83.166.711 | 6.74 |
| 2020 | 519.565 | 9.467 | 1.822,10 | 83.155.031 | 11.38 |
| 2021 | 488.498 | 17.499 | 3.582,21 | 83.237.124 | 21.02 |
| 2022 | 482.186 | 8.565 | 1.776,29 | 84.358.845 | 10.15 |

**Supplement Table 3 In-hospital mortality of ARDS with different classification**

| **ARDS classification** | **all**  **n** | **Survivors**  **n** | **non-survivors**  **n** | **mortality**  **[%]** |
| --- | --- | --- | --- | --- |
| unspecific | 2966 | 1576 | 1390 | 46.9 |
| mild | 3485 | 2359 | 1126 | 32.3 |
| moderate | 14476 | 8813 | 5663 | 39.1 |
| severe | 41911 | 17166 | 24745 | 59.0 |

**Supplement Table 4 In-hospital mortality of ARDS main diagnosis**

| **ARDS**  **main diagnosis** | **all** | **survivors** | **non-survivors** | **mortality** **[%]** |
| --- | --- | --- | --- | --- |
| Extrapulmonary sepsis | 53174 | 22417 | 30757 | 57.8 |
| Pneumonia | 52504 | 25473 | 27031 | 51.5 |
| COVID-19 | 21048 | 9785 | 11263 | 53.5 |
| Aspiration pneumonia | 9025 | 4417 | 4608 | 51.1 |
| Trauma | 1881 | 1322 | 559 | 29.7 |
| Pancreatitis | 2097 | 1012 | 1085 | 51.7 |
| Drown | 114 | 51 | 63 | 55.3 |

**Supplement Table 5 Comparison of characteristics between COVID-19 and non-COVID-19 ARDS**

|  | non-COVID-19  n [%] | COVID-19  n [%] | *P* Value |
| --- | --- | --- | --- |
| Total | 66.440 (75.9) | 21.048 (24.1) |  |
| Sex, F | 22.837 (34.4) | 6.744 (32.0) | <.0001 |
| Age, yr | 64 (52-74) | 66 (56-75) | <.0001 |
| EH index | 15 (8-23) | 11 (5-18) | <.0001 |
| Hospital LOS, d | 22.7 (11-41) | 19.9 (11-34) | <.0001 |
| ICU stay, d | 14 (5-26) | 14 (7-25) | <.0001 |
| *ARDS severity* |  |  |  |
| mild | 2.685 (4.0) | 547 (2.6) | <.0001 |
| moderate | 9.874 (14.9) | 3.836 (18.2) | <.0001 |
| severe | 25.656 (38.6) | 16.254 (77.2) | <.0001 |
| unspecific | 2.144 (3.2) | 410 (2.0) | <.0001 |
| *ARDS main diagnosis** |  |  |  |
| Extrapulmonary sepsis | 41.355 (62.2) | 11.817 (56.1) | <.0001 |
| Pneumonia | 40.378 (60.8) | 12.125 (57.6) | <.0001 |
| COVID-19 | 0 (0) | 21.047 (100) | <.0001 |
| Aspiration pneumonia | 8.640 (13) | 385 (1.8) | <.0001 |
| Trauma | 1.852 (2.8) | 29 (0.1) | <.0001 |
| Pancreatitis | 1.918 (2.9) | 179 (0.9) | <.0001 |
| Drown | 114 (0.2) | 0 (0) | <.0001 |
| *Comorbidity* |  |  |  |
| Congestive heart failure | 21.782 (32.8) | 4.974 (23.6) | <.0001 |
| Hypertension | 31.026 (46.7) | 12.556 (59.7) | <.0001 |
| Chronic pulmonary disease | 10.977 (16.5) | 3.157 (15.0) | <.0001 |
| Diabetes | 18.616 (28.0) | 7.355 (34.9) | <.0001 |
| Renal failure | 12.386 (18.6) | 3.850 (18.3) | 0.2539 |
| Malignancy | 7.438 (11.2) | 806 (3.8) | <.0001 |
| Obesity | 8.840 (13.3) | 4.090 (19.4) | <.0001 |
| *Ventilation* |  |  |  |
| Ventilation, yes | 64.355 (96.9) | 20.280 (96.4) | 0.0003 |
| Ventilation, d | 11.9 (5-24) | 12.4 (6-24) | <.0001 |
| *Assist device* |  |  |  |
| ECMO-VV | 6.925 (10.4) | 3.350 (15.9) | <.0001 |
| ECMO-VA | 1.747 (2.6) | 183 (0.9) | <.0001 |
| PECLA | 993 (1.5) | 31 (0.2) | <.0001 |
| ECOO2R | 105 (0.2) | 83 (0.4) | <.0001 |
| Mircoaxial pump | 138 (0.2) | 19 (0.1) | 0.0005 |

Data are presented as n (%) for categorical variables or median (interquartile range) for continuous variables. Group differences between categorical variables were tested for statistical significance with the χ2 test, and for continuous variables with Wilcoxon rank-sum test. *The different codes for the main diagnosis of ARDS were not mutually exclusive and were collected during the ICU stay involving ARDS.

ECMO-VA, venoarterial extracorporeal membrane oxygenation; ECMO-VV, venovenous extracorporeal membrane oxygenation; ECOO2R, Extracorporeal Carbon Dioxide Removal; PECLA, Pumpless Extracorporeal Lung Assist.

**Supplement Table 6 Age Distribution in COVID-19 and non-COVID-19 ARDS**

| **age**  **[yr]** | **ARDS cases**  **n (%)** | **non-COVID-19**  **n (%)** | **COVID-19**  **n (%)** |
| --- | --- | --- | --- |
| 18-19 | 451 (0.5) | 432 (0.7) | 19 (0.1) |
| 20-24 | 1.259 (1.4) | 1.173 (1.8) | 86 (0.4) |
| 25-29 | 1.490 (1.7) | 1.310 (2.0) | 180 (0.9) |
| 30-34 | 2.038 (2.3) | 1.738 (2.6) | 300 (1.4) |
| 35-39 | 2.617 (3.0) | 2.185 (3.3) | 432 (2.1) |
| 40-44 | 3.609 (4.1) | 2.967 (4.5) | 642 (3.1) |
| 45-49 | 5.285 (6.0) | 4.255 (6.4) | 1.030 (4.9) |
| 50-54 | 7.538 (8.6) | 5.861 (8.8) | 1.677 (8.0) |
| 55-59 | 9.420 (10.8) | 7.049 (10.6) | 2.371 (11.3) |
| 60-64 | 10.722 (12.3) | 7.741 (11.7) | 2.981 (14.2) |
| 65-69 | 11.305 (12.9) | 8.185 (12.3) | 3.120 (14.8) |
| 70-74 | 11.000 (12.6) | 8.162 (12.3) | 2.838 (13.5) |
| 75-79 | 10.313 (11.8) | 7.815 (11.8) | 2.498 (11.9) |
| 80-84 | 7.307 (8.4) | 5.222 (7.9) | 2.085 (9.9) |
| 85-89 | 2.618 (3.0) | 1.935 (2.9) | 683 (3.2) |
| 90-94 | 455 (0.5) | 354 (0.5) | 101 (0.5) |
| 95-110 | 61 (0.1) | 56 (0.1) | 5 (0.02) |

Data are presented as n (%) for categorical variables.

**Supplement Table 7 Complications and discharge of COVID-19 and non-COVID-19 ARDS**

|  | non-COVID-19  n [%] | COVID-19  n [%] | *P* Value |
| --- | --- | --- | --- |
| *complications* | | | |
| Renal replacement therapy | 27.204 (41.0) | 6.462 (30.7) | <.0001 |
| CPR | 12.180 (18.3) | 2.250 (10.7) | <.0001 |
| Pulmonary embolism | 3.194 (4.8) | 2.581 (12.3) | <.0001 |
| Myocardial infarction | 3.008 (4.5) | 606 (2.9) | <.0001 |
| Ischaemic Stroke | 2.631 (4.0) | 569 (2.7) | <.0001 |
| Intracerebral bleeding | 2.065 (3.1) | 754 (3.6) | 0.0007 |
| Arterial embolism/thrombosis | 1.551 (2.3) | 385 (1.8) | <.0001 |
| Anoxic brain damage | 2.138 (3.2) | 205 (1.0) | <.0001 |
| Cerebral oedema | 1.675 (2.5) | 298 (1.4) | <.0001 |
| CPR prior to hospitalisation | 1.101 (1.6) | 148 (0.7) | <.0001 |
| *discharge* |  |  |  |
| Home | 12.816 (19.3) | 4.350 (20.7) | <.0001 |
| Transfer | 12.730 (19.2) | 3.982 (18.9) | 0.4374 |
| Rehab | 5.594 (8.4) | 1.304 (6.2) | <.0001 |
| Care | 849 (1.3) | 144 (0.7) | <.0001 |
| Hospice | 41 (0.1) | 4 (0.02) | 0.0173 |
| Death | 34.409 (51.8) | 11.263 (53.5) | <.0001 |

Data are presented as n (%) for categorical variables. Group differences between categorical variables were tested for statistical significance with the χ2 test.

CPR, Cardiopulmonary resuscitation

**Supplement Table 8 Comparison of mortality and incidence in Acute Respiratory Distress Syndrome (ARDS) across various international studies**

| Author | Country | Study Period | Patient Population Characteristics | No. of Study Participants | Inclusion of COVID-19 (Yes/No) | In-hospital mortality  [%] | Incidence |
| --- | --- | --- | --- | --- | --- | --- | --- |
| Heinicke et al. | Germany | 2008-2022 | ARDS,  >18 years | 87.488 | Yes | 32.3 (mild)  39.1 (moderate)  59.0 (severe) | 7.10/100.000 inhabitants (cumulative) |
| Bardají-Carrillo et al. | Spain | 2000-2022 (without 2016) | mechanically ventilated ARDS,  >18 years | 93.192 | Yes | 40–45 | 2.96 - 20.14/100,000 population-years |
| Karagiannidis et al. | Germany | 2019-2022 | mechanically ventilated,  >18 years | 1.003.882 | Yes | 43.3 (total)  53.7 (COVID-19)  42.6 (non-COVID-19) | - |
| Papazian et al. | France | 2017 | ARDS,  >18 years | 12.846 | No | 51.1 | 6.7- 51.9; total =24.59 (per 100,000 person-years) |
| LUNG SAFE | International | 2014 | ARDS,  >16 years | 3.022 | No | 34–46 | 0.27 to 0.57 cases per ICU bed per 4 weeks and percentage of ICU admissions |

ARDS, Acute Respiratory Distress Syndrome

**Supplement Table 9 Summary of the Interrupted Time Series (ITS) Model for ARDS Incidence (GLS Regression)**

| Variable | Estimate | Std. Error | *t* value | *p*-value |
| --- | --- | --- | --- | --- |
| Intercept | 3.49 | 0.18 | 19.57 | < 0.001 |
| Time (pre-COVID) | +0.26 | 0.03 | 9.81 | < 0.001 |
| Intervention (2020) | +7.04 | 1.54 | 4.57 | < 0.001 |
| Post-intervention trend | +0.64 | 0.80 | 0.81 | 0.437 |

*ARMA(1,1) model: AR(1) = –0.86, MA(1) = –1.00; AIC = 55.5, residual SE = 3.04*

**Supplement Table 10 Summary of the Interrupted Time Series (ITS) Model for ARDS In-Hospital mortality (GLS Regression)**

| Variable | Estimate | Std. Error | t value | p-value |
| --- | --- | --- | --- | --- |
| Intercept | 52.48 | 0.88 | 59.93 | < 0.001 |
| Time (pre-COVID) | –0.14 | 0.12 | –1.23 | 0.245 |
| Intervention (2020) | +1.89 | 1.65 | 1.15 | 0.276 |
| Post-intervention trend | +0.37 | 0.75 | 0.49 | 0.635 |

*ARMA(1,1) Modell: AR(1) = 0.41, MA(1) = –0.10; AIC = 53.6, residual SE = 0.95*
